# Circular Economy and the Changing Geography of International Trade in Plastic Waste

**DOI:** 10.3390/ijerph192215020

**Published:** 2022-11-15

**Authors:** Enru Wang, Changhong Miao, Xiaofei Chen

**Affiliations:** 1 Department of Geography and Geographic Information Science, University of North Dakota, Grand Forks, ND 58202, USA; 2 Key Research Institute of Yellow River Civilization and Sustainable Development, Collaborative Innovation Center on Yellow River Civilization Jointly Built by Henan Province and Ministry of Education, Henan University, Kaifeng 475001, China; 3 College of Geography and Environmental Science, Henan University, Kaifeng 475004, China

**Keywords:** plastic pollution, circular economy, plastic waste, international trade, value, North–South dichotomy, sustainable development, environmental justice, Basel Convention

## Abstract

Plastic pollution has become a major environmental concern worldwide. As the circular economy is increasingly seen as a means for achieving sustainable development, it is imperative to promote the more efficient use of plastics worldwide. An integral part of the circular economy model, trade in waste, and the scrap for recovery is a part of the solution to achieve sustainability. This paper studies the changing geography of the international trade in plastic waste. It reveals increasingly complex patterns of the transboundary trade in plastic waste over more than two decades. The movement of plastic waste from high-income countries to developing nations has been the largest flow, but trade flows of other directions turn out to be significant. The findings of the paper debunk the North–South or core–periphery dichotomy that is embedded in the international environment justice tradition (including the ecologically unequal exchange theory) as well as in international environmental regulatory regimes such as the Basel Convention. The paper contributes to the discussions about value that are central in political economic approaches to global trade (e.g., the global value chain and global production network) by demonstrating the relative, spatial, and dynamic nature of the concept. As the transboundary trade in plastic waste has exacerbated pollution and marine litter in some major receiving countries, it needs to be better monitored and regulated to ensure it is conducted in a transparent and environmentally sound manner. The paper also explores several policy measures that could help tackle the plastic pollution crisis and achieve sustainable development.

## 1. Introduction

Plastic has become one of the most widely used materials in industry and our daily life. After World War II, the production of synthetic plastics skyrocketed from 1.7 million tons in 1950 to 368 million tons in 2019 [1,2]. The plastics industry has become a major part of the world economy. In the United States, the plastics industry is the eighth largest industry and the third largest manufacturing industry, accounting for over one million jobs and USD 432 billion in shipments in 2019. If suppliers were included, the industry created 1.5 million jobs in the nation [3]. Due to their versatility and innovation capacity, plastic materials offer solutions to a wide variety of needs in innumerable products and applications. The global plastic products market grew from USD 796.87 billion in 2015 to USD 1008.5 billion in 2019, with an annual growth rate of 6.01%. After a slump in 2020 due to the COVID-19 pandemic, it is expected to grow USD 1258.1 billion by 2025, at an annual growth rate of 6.19% [4].

The rising consumption of plastic has led to an accelerated generation of plastic waste. The global trade in plastic waste has exploded since the early 1990s, growing into a multi-billion dollar business. With plastic pollution, especially marine litter, becoming a major environmental concern worldwide, people have started questioning the role that the global trade in plastic waste plays [5].

A burgeoning literature has emerged on the international trade in waste and scrap. The mainstream of the literature tends to perceive the waste as the throwaway that consumer societies of the Global North have dumped on the people and environments of the Global South. Framed by the global environmental justice (GEJ) paradigm, this stream of studies, especially those under the umbrella of the ecologically unequal exchange (EUE) theory, often treats international trade in waste as a one-directional flow of waste that externalizes the environmental costs of the Global North using the Global South as the waste sink [6]. In recent years, another stream of research has analyzed the international trade in waste from the perspective of global economic chains and networks, notably global value chain (GVC) and global production network (GPN) analyses. The myriad writings on trade in post-consumer wastes include those concerning ships, automobiles, clothing, and so on. Electronic waste, or e-waste, has received much publicity as a rapidly growing problem. Surprisingly, however, international trade in plastic waste has not received much attention even though “trade plays a central role in plastic pollution and in the global plastics economy” [7]. Since the 1990s, the shipment of plastic waste from developed to developing countries has been increasing. The case of international plastic waste trade suits well to reveal the relevant power asymmetries in the global economy that have been examined in both streams of political economic studies (i.e., EUE and GVC/GPN).

As the circular economy (CE) is being recognized as a solution to sustainable development [8], the concept “new plastics economy” is being proposed and incorporated into the CE strategy to address the environmental problems caused by the production, consumption, and disposal of plastics [9]. The new plastics economy aims to reduce waste, maximize value, and use plastic efficiently. Central to this notion is recycling, as well as the movement or redistribution of recycled goods for the purpose of reuse. Thus, it is imperative to study the trade of plastic waste and its role in the future CE.

In this paper, we aim to reveal the evolving patterns of the global trade in plastic waste and to explore the future of plastic waste trade. We hope to contribute to the debate on issues related to the global trade in waste in general and plastic waste in particular: does global waste trade represent toxic colonialism? An impeccable search for new value? Or a plausible move toward sustainability? By doing so, the paper provides links to the EUE and GVC/GPN concepts by offering evidence of the power asymmetries that drive the dominant movement of plastic waste. Meanwhile, by revealing a complex geography of plastic waste trade flows, the study reexamines some stylized forms of thinking associated with the EUE theory (such as the North–South or core–periphery dichotomy). We will also discuss the concept of value that is central in the GVC/GPN approaches and use the concept to explain the changing geography of plastic waste trade. The paper will contribute to the discussion on CE as one potential solution to the plastic pollution crisis that the world faces.

The rest of the paper is organized as follows: Section 2 reviews the literature on the international trade in waste and discusses the concept of CE. Section 3 introduces the data and analysis methods. The paper then proceeds to Section 4 to examine the temporal and spatial patterns of the global trade in plastic waste. A case study of China, a leading importer of plastic waste for over two decades, is provided in this section. The concluding section summarizes the findings of the study and discusses the policy options for tackling the plastic pollution crisis.

## 2. Literature Review

In this paper, we situate our work within three streams of literature that relate to international trade, including a recent discussion of the CE and two political–economic traditions: the EUE theory and the GVC/GPN approaches. We seek to make several contributions to the literature on the international trade in waste. First, we will contribute to the discussion of the CE as a potential solution to the current plastic pollution crisis. Second, we debunk the North–South (or core–periphery) dichotomy that is embedded in the EUE theory as well as in international environmental regulatory regimes (such as the Basel Convention) by revealing an increasingly complex pattern of plastic waste trade at the global scale. Third, the study will make a contribution by expanding the conceptualization of the value that is central in the GVC and GPN approaches by demonstrating the relative, spatial, and dynamic nature of the concept.

### 2.1. Circular Economy as a Move toward Sustainability

The drawbacks of the plastic economy are becoming increasingly apparent. Today, 99% of plastics are derived from fossil fuels, consuming about 6% of global oil. If the current trends in oil consumption and plastic production continue, it is projected that by 2050, the consumption of oil by the plastics sector will account for 20% of the total oil consumption in the world [10]. As the largest end-use market segment, packaging account for over 40% of the total plastic usage. It is also the largest source of plastic waste. Each year, only 14% of all plastic packaging is collected for recycling after use, with vast quantities escaping into the environment, resulting in a loss of USD 80 to 120 billion per year [11].

Compared to the economic costs, the environmental impacts of plastics consumption are more detrimental. Between 1950 and 2015, an estimate of 8.3 billion tons of plastics were produced, from which 6.3 billion tons of plastic waste were generated. Among the wastes, 9% and 12% were recycled and incinerated, respectively, while 79% ended up in landfills and nature. If the current production and waste management trends continue, the amount of plastic waste entering landfills or the natural environment will likely double by 2050 [12]. The plastic pollution of ecosystems is becoming a scourge. It is estimated that 100 million tons of plastic have accumulated in the world’s oceans. Yet, approximately 8.8 million tons of plastics are entering the oceans each year [13]. If the current trend continues, there could be more plastic than fish by weight in the oceans by 2050 [11]. The plastic pollution of oceans has led to the endangerment of species, as plastics pose major physical and chemical threats to aquatic life due to ingestion and/or entanglement. Unless dramatic measures are taken to reverse the trend, the dire warning “The oceans are drowning in plastic!” is becoming a reality [14]. In addition to plainly visible pieces of plastic debris, microplastics, which come from the degradation of plastic debris and many other sources, are entering the environment (including soils and drinking water) and end up in the food chains, raising further health concerns to humans and animals.

The heightened awareness of the plastic pollution crisis calls for a CE model to mitigate the environmental externalities of plastic production and consumption. The term “circular economy” was first coined in 1990 by Pearce and Turner in their now widely cited book that explored the relationship between the economy and the environment [15]. The concept has received increased attention in recent years from researchers and practitioners. Recognizing the CE as an “irreversible, global mega trend”, the European Union (EU) unveiled a new Circular Economy Action Plan as an agenda for sustainable growth in 2020 and adopted the CE as an essential strategy for accomplishing of the goals set in the European Green Deal [16]. An increasing number of countries have incorporated the CE into their government action plans. The concept of the CE has roots in the “3R” framework: reduce, reuse, and recycle [15]. Defining the concept, however, has not reached a consensus after 30 years of development [17]. Despite the momentum that the CE is gaining, the concept means many different things to different people. Kirchherr et al. (2017) gathered 114 CE definitions and coded them on 17 dimensions [18]. Their findings indicate that the CE concept is most frequently depicted as a combination of reduce, reuse, and recycle activities. Many other scholars also appear to agree that reduce, reuse, and recycle are the most commonly used CE strategies [19,20,21]. Indeed, the CE is a diverse bundle of ideas [22]: one of its core principles is the recycling of waste (or used goods) for reuse through remanufacturing/refurnishing. Improving the recovery rate of recyclable materials and facilitating the movement or redistribution of recycled goods are two important (first) steps of the process. As Gregson et al. (2015) argued, the global trade in waste is a key means by which materials are recovered for their reuse and recycling [23].

### 2.2. The Ecologically Unequal Exchange Theory and the Basel Convention

The EUE theory is grounded in Wallerstein’s (1974) world-systems perspective and the pioneering work of Bunker (1984, 1985) and other critical development scholars [24,25,26]. It centers attention on the extraction of natural resources and materials from less developed countries (and regions) to developed countries (and regions) and the export of waste disposal and hazardous production activities from the latter to the former. Through the unequal exchange that makes up their relationships, developed countries shift environment costs by externalizing social and ecological burdens of extraction, production, and consumption to less developed countries. International trade in waste has often been framed as colonial dumping or toxic colonialism [27,28,29]. The EUE theory highlights that underlying such unequal flows are systems of unequal political and power relations. Nations in the periphery are structurally positioned as both a tap into resources and a sink for waste within the global economic system to meet the needs of the core industrial centers [30,31].

The EUE theory provides an important perspective that enables analyses of the unequal power structure and trade relationships among nations and facilitate a better understanding of many global environmental problems. As Hornborg and Martinez-Alier (2016: 329) argue, the EUE “is an underlying source of most environmental distribution conflicts in our time.” [32] Treated as a theory of the GEJ movement, the EUE theory presents an approach linking GEJ research with a global structural perspective [31,33]. In recent years, there has been much renewed interest in the EUE, but proponents of the EUE theory still overwhelmingly concentrate in the field of environmental sociology. Another challenge facing the EUE approach comes from its assumption based on the dichotomous core–periphery structure that underpins the orthodox world-system theory. This geographical core–periphery dichotomy in terms of capital accumulation and environmental burden distribution is insufficient, as ecological distribution conflicts may also occur among the nations both within the Global North and within the Global South [30,34].

The North–South dichotomy is also embedded in the Basel Convention, or the Basel Convention on the Control of Transboundary Movements of Hazardous Wastes and their Disposal, a United Nations Convention based in Geneva. Considered to be the most comprehensive global environmental treaty governing the classification and cross-border movement of hazardous wastes and other wastes [35], the Basel Convention came into force in 1992 and has a nearly universal membership with 187 parties. Adopted in 1995, the Ban Amendment to the Basel Convention defines two groups of countries: Annex VII countries consisting of members of the European Union (EU) and the Organization of Economic Cooperation and Development (OECD) as well as Liechtenstein (i.e., mostly high-income countries), and non-Annex VII countries that include all other signatories to the Basel Convention. The Ban Amendment seeks to prohibit transboundary shipments of hazardous waste from Annex VII to non-Annex VII signatories, under the assumption that Annex VII countries have the technological and regulatory capacity to manage hazardous waste, while non-Annex VII countries lack it [36].

Several studies have evaluated the limitations of the Basel Convention and the Ban Amendment [37,38]. One of the controversial aspects of the Basel Convention is that it embraces a geographical imaginary that is fundamentally based on the “North–South” or “core–periphery” constellation and seeks to prohibit the shipment of hazardous waste from the Global North to the Global South [36,39]. This geographical imaginary overlooks the increasingly complex patterns and nature of the global trade in waste (including plastics waste). Another criticism of the Basel Convention is that plastic waste does not receive adequate attention in the current guidelines. The Basel Convention classifies plastic waste into “hazardous wastes” and “other wastes”, with much of the plastic waste falling into the latter category. The Convention obligates parties to manage the generation and cross-border movement of such wastes. Moreover, like many other international environmental agreements, the Basel Convention lacks a formal enforcement mechanism and provisions for formal legal sanctions for noncompliance [40].

Regardless, the Basel Convention provides a framework for countries to manage their own wastes and control the illicit movement of wastes across borders. The principle of prior informed consent (PIC), the “keystone” of the Basel Convention and other major international environmental agreements [41], requires the exporting party to obtain an agreement from the prospective parties to accept an import or allow the transit of intended transboundary movements of hazardous wastes, before allowing the shipments to depart the country. Prior to 2019, most plastic wastes were classified as “other wastes” which were not subject to the PIC procedure. The 2019 meeting of the Basel Convention passed a series of amendments, among which the most impactful change was the addition of listings A3210 and Y48 to Annex VIII and Annex II, respectively. The two listings bring under the “Categories of Wastes Requiring Special Consideration” most plastic wastes, thus making them subject to the PIC procedure, effective by 2 January 2021 [42].

Like other types of waste, plastic waste has been predominately flowing from developed to developing countries. The central argument of the “Global South as dumping sites for wastes” in the EUE theory and the GEJ movement likely find some support in the case of plastic waste. Nonetheless, this study contributes to the EUE literature in two ways: (1) the complex geography of plastic trade observed in this study will expose the limitations of some stylized forms of thinking associated with the EUE theory, such as the North–South dichotomy, which is also embedded the Basel Convention; (2) the discussion of the value of using China as an example will show that the ecological colonialism thesis may be too simplistic.

### 2.3. The GVC/GPN Approaches to International Waste Trade

The second stream of the literature is built on conceptually related perspectives, the GVC and the GPN perspectives. The origins and evolution of the GVC/GPN approaches have been reviewed in depth elsewhere [43,44,45]. Often treated as a family of theories [46,47], the two conceptualizations could be understood as similar successors to radical theories of global political economic theories, such as dependency theory and world-systems theory [43,48].

The GVC/GPN frameworks are powerful tools to understand the trade and flow of goods and services on a global scale. By mapping the relations of production, they have demonstrated an effectiveness in revealing the creation, capture, and distribution of value in commodity chains or production networks. Meanwhile, these approaches also have limitations in studying the international trade of waste. These approaches, especially the GVC approach, have been criticized for their linear assumptions [46,47,49]. In the GVC approach, a value chain is “the full range of activities which are required to bring a product or service from conception, through the intermediary phases of production, delivery to final consumers, and final disposal after use” [50]. The disposal of waste is simply the end of the chain. The GPN approach attempts to address the linearity problem by incorporating network configurations and considering material flows in all directions. Recent work has demonstrated the possibilities for studying the South–South trade using the GPN approach [51,52]. Nevertheless, while GPN studies highlight the multi-directional flows of knowledge and capital, there is still “a tendency to preserve a linear sense of material flows” [46]. Both frameworks have not seriously considered the circular flow of goods and materials in the global economy. Another related limitation is that most of the GVC/GPN literature fails to account for the post-consumption activities and the value of end-of-life goods. In the GVC/GPN frameworks, value is treated as an intrinsic characteristic of objects and materials, representing the portion of the final price that is created, enhanced, and/or captured by a given actor or location along a value chain or in a production network [53]. The unidirectional flow of goods or the sequential transformation of materials usually ends with the consumption or purchase of goods. End-of-life goods are virtually treated as valueless. Despite a stated interest by GVC/GPN scholars in the post-consumption “after-lives” of products and services [44,49,50], the economic lives of used goods have been largely ignored in these literatures [46].

Alongside proliferating discussions of GVCs and GPNs has emerged a small but growing literature on waste, with studies concerning the disassembly of ships [23,46], automobiles [54,55], clothing [46,56], and e-waste [53,57,58,59,60,61]. With few exceptions [46,53], the new literature has not explicitly discussed the dynamic and spatial nature of value.

Compared with other types of waste or end-of-life product, such as e-waste, scrap metal, and old automobiles, plastic waste is less valuable but more voluminous, with more profound environmental and public health impacts. Thus, it is important to study the trade flows of plastic waste. Our paper will contribute to this new literature and the broader GVC/GPN literature by revealing the breadth of the global network of plastic waste trade. As a significant amount of value is recovered with massive flows of plastic waste, this study will show that end-of-life plastic products are not valueless. Plastic waste deserves more attention in future GVC and GPN studies as post-consumption value needs to be accounted for. Through the case of China (to be discussed in Section 4), the study will demonstrate the relative, spatial, and dynamic nature of value, a concept that is central in the GVC and GPN analyses.

In developed countries, due to the high cost of recycling and recovering processes (e.g., high labor cost, strict environmental regulations, and related high environmental management cost), plastic waste, if not collected to be exported overseas, would largely be treated as garbage with little value to recover and reuse, ending up in landfills. By contrast, in many developing countries, especially in newly industrializing economies, a variety of factors (e.g., cheap labor, lax environmental regulations, and the growing need for resources) contribute to a booming recycling industry and create opportunities for important plastic and other waste. With the interest and ability to rekindle value in recalcitrant waste materials and transform them into resources, these countries treat plastic waste and scraps as valuable resources for industries. In this respect, value is a relative concept as waste means different things in different countries and carries different weights in their economies. Moreover, the ability to rekindle value from waste and used things is geographically uneven [23,46]. That differentiated ability, which is constrained by economics (such as costs, especially labor costs, of recycling), societal factors (i.e., societal acceptance of environmental and health risks associated with processing wastes), and legal factors (environmental regulations and enforcement) drives the trades in used materials. Thus, it is necessary to treat value as a spatial concept in global recycling networks. Value is also a dynamic concept as it changes over time and with circumstances. Like other commodities, the economic value of waste and scraps–reflected in the price–fluctuates on the global market. Similarly, the value of waste and scraps, which can be considered as “importance” or “worth” [62], may change when a country’s domestic circumstances change (as shown in the China example in Section 4).

## 3. Materials and Methods

To trace the pattern and measure the volume of the international trade in plastic waste, we used data which were extracted from the United Nations (UN) Comtrade database [63]. The harmonized system (HS) code 3915 (waste, parings and scrap, or polymers of ethylene) was used for the import and export data of various polymer categories and major product categories containing plastic. Specifically, the 3915 category consists of several subcategories, including 391,510 (polyethylene waste or scrap), 391,520 (polystyrene waste or scrap), 391,530 (polyvinyl chloride waste or scrap), and 391,590 (plastics waste or scrap of other plastics).

In the reporting scheme of the UN Comtrade database, the reporters—countries or territories that report the trade data—and trading partners are countries in most cases. In some cases, however, they represent other types of territories (for example, the “US Virgin Isds [Islands]”; “China, Hong Kong SAR”; “Other Asia nes [not elsewhere specified]”). The data used in mapping the flows of plastic waste between territories and regions were the import data reported by the trading territories. It is believed that import data are more accurate than export data [64], because a territory is more likely to accurately report their imports rather than exports. As a cautionary note, our analysis of the export data in comparison with the import data also suggests that export data in the UN Comtrade Database may not be accurate.

To analyze the flows of plastic waste among different types of countries and territories, including the flow from developed to developing countries as emphasized in the EUE literature and GVC/GPN approaches to international waste trade, we adopted the delineation of countries and territories in the Basel Convention (i.e., Annex VII countries versus non-Annex VII countries).

To visualize the evolving patterns of the global trade in plastic waste, we employed an approach similar to what was used in the work by Lepawsky [36,65]. Gephi, a free and open-source graphic visualization and network analysis platform [66], was used to produce cartograms depicting the cross-border flows of plastic waste. While trading territories are represented as “nodes”, plastic waste trade flows are depicted as “edges”. The size of a given node is proportional to the number of trade transactions reported by all the other nodes, whereas the thickness of an edge is proportional to the volume or weight (in kilograms) of a given transaction between two nodes relative to all the other transactions in the graph. The edges were curved to indicate the direction of the transaction from one node to another in a clockwise direction. Transparency (40%) was used to show thinner edges that otherwise would be covered by overlapping thicker ones. The nodes representing Annex VII territories and the edges which originated in Annex VII territories were colored in sienna, whereas the nodes representing non-Annex VII territories and the edges which originated in non-Annex VII territories were colored in steel blue. A georeferenced trade network was created by assigning each node geographic coordinate (based on the latitude and longitude of the territory’s capital). Mercator projection layout was used.

China has been one of the most important players in the international trade of plastic waste for over two decades. The country introduced a strict ban on waste imports, including waste plastics, with a far-reaching impact globally. In Section 4, we provide an analysis of China as a case study to shed light on the relative and dynamic nature of value in plastic waste.

## 4. Results

In this section, we first provide a general overview of the global trade in plastic waste in relation to the global production of primary plastic. We then reveal China’s role in the international waste trade by applying the notion of value and demonstrating the relative, spatial, and dynamic nature of the concept. The last part of the analysis reports the spatial patterns of international transboundary flows of plastic waste during 1996–2019. The main findings that emerge from the analysis lead us to reexamine some stylized forms of thinking, such as the core–periphery dichotomy inherent in the world systems theory and its application (e.g., the EUE theory).

### 4.1. Global Trade in Plastic Waste over Time: An Overall Picture

**Figure 1:**
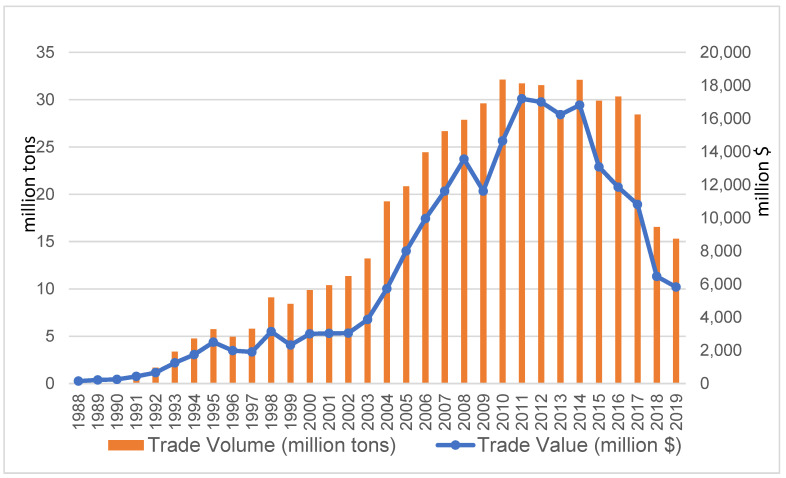
Global trade in plastic waste over time.

Throughout the 1980s, plastic recycling was mainly limited in a small number of developed nations in North America and western Europe, before it became more commonplace in the 1990s. As a result, there was a very limited trade in plastic waste in the 1980s. The UN Comtrade data show that in 1988, the total volume (import plus export) of the global trade in plastic waste was less than 0.32 million tons, with a total trade value of USD 149 million. After a steady growth in the 1990s, the global trade in plastic waste skyrocketed in the 2000s. In two decades, the plastic waste trade became a large and lucrative business. From 2001 to 2011, the trade volume on average increased by almost 12% annually, while the trade value increased by 19% each year (Figure 1). The total trade volume of plastic waste peaked in 2010 at 31.2 million tons, whereas the total trade value peaked a year after in 2011 at USD 17.2 billon. The trade volume and value dipped in 2013 when China launched a 10-month campaign known as “Operation Green Fence” which was aimed at curtailing the importation of contaminated recyclables and waste. After a rebound in 2014, the global trade in plastic waste declined since then, with China putting more restrictions on importing solid waste. It plunged in 2018 after China decided to ban the import of 24 categories of solid waste.

In the 1990s, Hong Kong, a former British colony that was returned to China as a special administrative region (SAR) in 1997, was the leading importer of plastic waste in the world. In 2000, China (referred to as mainland China in the analysis) became the largest importer and retained that position until 2017, with Hong Kong SAR being the second largest. Other major importers that made to the “top five” list most frequently included the USA, the Netherlands, Belgium, Denmark, and Canada. However, compared to mainland China and Hong Kong SAR, the number of imports by these countries was significantly smaller. In recent years, industrializing economies in Asia, such as India, Malaysia, and Thailand, have seen an increase in their import of plastic waste. Malaysia emerged as the largest importer in 2018 after China imposed a ban on importing plastic and other solid waste. From the early 1990s to 2017 (except for 2013), Hong Kong was the largest exporter of plastic waste, but it had largely served as a “collector” of plastic waste from around the world and reexported much of its massive imports to mainland China. Other major exporters of plastic waste on the global market included the USA, Japan, the Netherlands, Germany, France, the UK, and Belgium. It is worth nothing that for some large high-income economies, such as the USA, Japan, Germany, France, the UK, and others, their export data appear to be underreported. If we consider the sum of all other countries’ imports from a country as a reasonable estimate of the country’s actual export, for these high-income countries, their reported export numbers appeared to be significantly smaller than their actual exports, varying from 90% to less than 30% depending on the year of the reporting. As Lepawsky (2015) explained, a trading territory tends to be more careful about what is imported into its borders than what is exported out (e.g., an exporter is less likely to report an illicit trade) [65].

### 4.2. China and International Trade in Plastic Waste

In order to understand the evolution and changing geography of the international trade in plastic waste, special attention needs to be paid to China, which has been one of the most important players in the international trade of waste (including plastic waste) for over two decades. After emerging as a major importer in the 1990s and rising to the top importer in 2000, China’s import volume of plastic waste increased by 14.6% annually during 2002–2012, while its import value increased by 28% each year. During 2006–2016, China alone imported about a half of the world’s plastic waste. Indeed, in line with the EUE theory, the country became a global dumping ground or waste sink. That said, this status was more or less self-imposed. Beginning in the 1990s, the demand for raw materials in China exploded to fuel the rapid economic growth. A continuous massive inflow of rural migrants into cities and towns provided cheap labor for the labor-intensive recycling industry, which in return created millions of jobs in cities. Many “plastic villages” sprung up, most of which are located near cities, building their economy almost entirely on recycling plastic waste. The plastic recycling industry became a highly lucrative business. By 2017, the plastic recycling sector was valued at CNY 108.1 billion (about USD 15.8 billion), only after the iron and steel recycling and non-ferrous metal recycling sectors [67]. The huge demand and a rapidly growing industry were, of course, not the only factors that contributed to China’s emergence as the center of the global waste trade. Compared with developed countries, environmental regulations in China were not adequately enforced (until recent years), which made it easier for international waste to be imported. For these reasons, China had the interest and ability to rekindle value in recalcitrant waste materials and transform them into resources.

China’s example also illustrates that value is also a dynamic concept as it changes over time and with circumstances. The country’s seemingly insatiable appetite for imported plastic waste has dwindled in recent years. Plastic scarps imported into China for recycling, often mixed with other waste and hazardous elements including medical waste, are usually collected in open dumping grounds or landfills. Much of the plastic waste treatment takes place in the informal sector. Due to lack of oversight, workers are exposed to hazardous materials and toxic air. The plastic scraps collected from landfills require a large amount of water to clean, which are often processed in primitive, family-owned, or other small workshops with no facilities to treat wastewater before it is discharged into local rivers and streams. Recycling only makes a small dent in the amount of plastic waste generated each year. While some is incinerated or goes to landfills, a large amount is left at open dumping sites and washed to the surrounding environment or burned on the site, causing severe pollution to the land, water, and air. Citing these environmental and health concerns, China launched the National Sword campaign in 2017, implementing an outright ban of 24 categories of solid waste including tires, textiles, plastic, and glass (unless recyclables were at least 99.5 percent pure) and limiting the importation of other waste, which started in 2018.

In addition to the widely cited environmental and health concerns, a less examined factor for China’s changing attitude toward scrap imports might have to do with economics. Over time, China has greatly expanded its plastic production capacity. For over two decades during 1996–2017, the production of primary plastic averaged an annual growth of nearly 14% [68] As a result, plastic production in China, especially the production of low-end plastic materials, outpaced demand in recent years [69]. Meanwhile, resin producers worldwide responded to the production of low-price oil and natural gas by adding a tremendous new capacity, keeping prices for virgin resins lower than those for recycled plastics [70]. As China started to have the overcapacity problem in plastic production, the changed economic circumstances reduced the need for importing plastic scraps.

Taken together, China’s regulatory shift of in its waste import policy was likely a result of a combination of environmental concerns and economic considerations. Even though most of the waste and scraps feeding China’s massive and inadequately regulated recycling industry comes from domestic sources, restricting waste imports understandably was a first easy step to take. Plastic scraps, largely treated as valueless waste in developed countries but once regarded as valuable resources in China, are being rejected in the country due to their diminished value. It is worth pointing out, though, that China is not unique or the only case of this. Plastic waste imports by India, for example, increased by more than 9 times during 1996–2013. From 2014, the country’s import started to level off and decline. Following China, India started to ban the import of plastic waste from August 2019. Other countries in Southeast Asia, such as Malaysia, the Philippines, and Thailand, are following a similar path. After increased import for years, especially after becoming the new hotspot destinations in the last two years for western countries to reroute their export of plastic waste, these countries have seen the massive inflow of plastic waste and scraps that are beyond their needs, overwhelming their environment. Therefore, they are reportedly sending an increasing number of shipping containers back to their countries of origin.

### 4.3. Changing Geography of Global Trade in Plastic Waste

**Figure 2:**
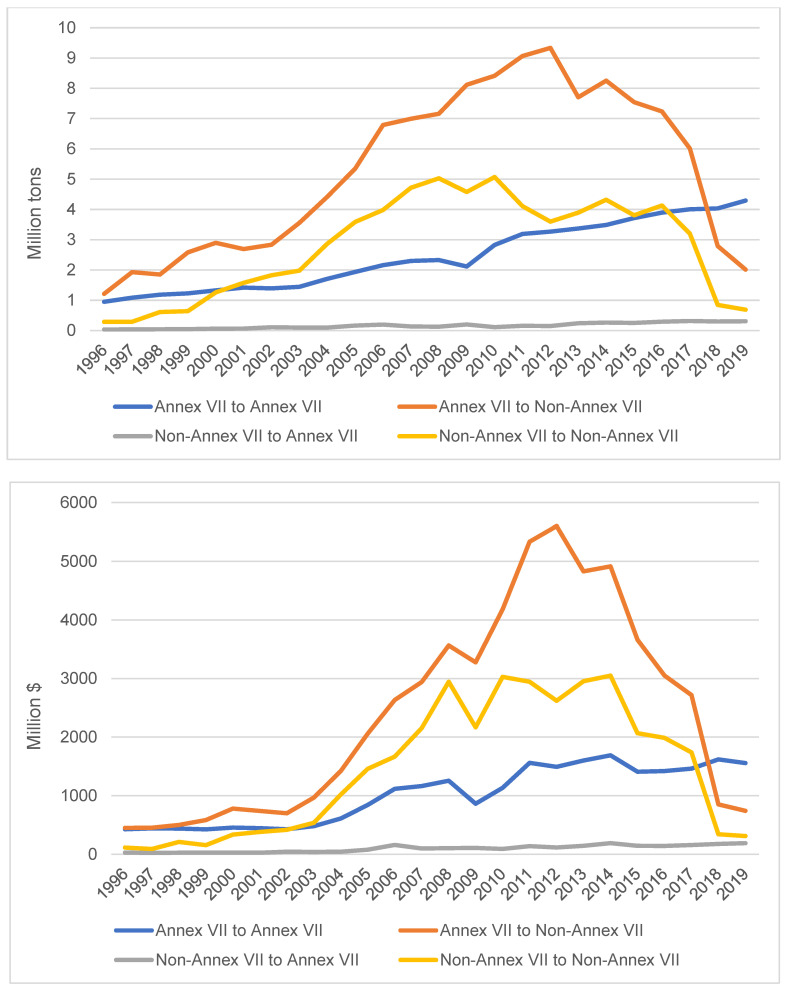
Trade flows of plastic waste by weight (**top**) and by value (**bottom**). Note: Annex VII countries consist of EU and OECD members as well as Liechtenstein; non-Annex VII countries include all other signatories to the Basel Convention.

By examining the flows of plastic waste between and within Annex VII and non-Annex VII territories, several observations can be drawn (Figure 2). First, the movement of plastic waste from Annex VII to non-Annex VII territories had been the largest flow until 2018. High-income countries have been exporting plastic waste to countries that may not have the capacity to manage them in an environmentally sound manner. Nevertheless, the dominance of the plastic waste flow from the core to the periphery may not be as strong as is often assumed. From 2018, the flow from Annex VII to non-Annex VII territories is no longer the largest stream of the plastic waste trade. Second, trade flows in other directions, which are often overlooked in the EUE literature and other GEJ traditions, turned out to be significant. The plastic waste trade among Annex VII territories has been rising steadily and it has become the largest flow since 2018. The plastic waste trade among non-Annex VII territories also increased quickly during the 1990s and 2000s. Taken together, these two streams of the plastic waste trade exceeded the trade flow from Annex VII to non-Annex VII territories for most years during 1996–2019 (except for the period 2009–2015). One may expect that if the international trade in plastic waste continues in the future, more would take place within high-income economies and within developing economies, respectively. Third, an interesting finding is that there has been a small but growing flow of plastic waste from non-Annex VII to Annex VII territories. By 2014, this trade flow exceeded the total imports of the global trade in plastic waste in 1990. This finding does not fit well with the dominant narrative of the international waste trade (e.g., from the Global North to the Global South). Nevertheless, it is not surprising given that a similar pattern has been found to exist in the e-waste trade (Lepawsky, 2015) [65].

The visualization of the plastic waste flow offers several snapshots of the global plastic waste trade for the period 1996–2019 (1996, 2006, 2016, and 2019, Figure 3a–d). An examination of these maps reveals some distinct geographical patterns and their evolution over time. In 1996, the USA was the single largest source of plastic waste in terms of both the volume traded and the number of territories reporting imports from the country. Germany and Japan were closely ranked as the second and third largest sources, respectively. EU members, including the Netherlands, France, Belgium, the UK, and Italy, were also significant sources. With regard to the importers, the single largest recipient of plastic waste imports was Hong Kong, which accounted for 47% of the total imports in 1996. Among its massive import, 43% came from two countries, the USA and Japan. One surprising finding relates to the USA. The country recorded not only the second largest volume of imports but also the single largest number of trading partners. Nearly two thirds of the country’s imports came from three countries: Canada, Mexico, and Germany. China was the third largest importer, with the USA, Japan, and Hong Kong being the obvious top three sources. Other importers that appeared on the top 10 list included the Netherlands, Italy, Canada, Belgium, Germany, Mexico, and Ireland. Among them, Canada and Mexico primarily imported from the USA, whereas other countries mainly traded with one another within the EU.

In 2006, China became the largest recipient of plastic waste, with Hong Kong SAR being the most important source, followed by Germany, the Philippines, the USA, Spain, and Japan. China’s plastic waste trade literally reached over the world, with imports coming from 110 countries and territories. Hong Kong SAR was the second largest importer, receiving plastic waste from a very large number of sources, especially from Japan, the USA, the UK, the Netherlands, and Germany. As the third largest importer, the USA also received plastic waste from a large number of countries and territories, even though the country imported only one tenth of that which China imported. Other major receivers mainly included EU countries, such as the Netherlands, Germany, Belgium, and Italy. In addition to Hong Kong SAR and the USA, other major exporters included Japan, Germany, the UK, the Netherlands, Belgium, and France. These EU countries increased their exports to China and Hong Kong SAR, but trade within the EU remained important.

In 2016, China retained its dominance as the leading importer of plastic waste and scraps. While Hong Kong SAR remained the mainland’s largest trading partner, other large-volume flows came from Japan, the USA, Thailand, Germany, Belgium, the Philippines, and Australia. In part, the “middleman” role that Hong Kong had played between China and the rest of the world declined. In addition to China and Hong Kong SAR, other major receivers included the Netherlands, Germany, the USA, Belgium, etc. Industrializing countries such as Malaysia and India also imported a large amount. The USA, based on the sum of the total imports from the country, surpassed Hong Kong SAR to become the largest exporter of plastic waste, a pattern that would be concealed by the self-reported export data. Other major sources included Japan and traditional exporters from the EU (e.g., Germany, the UK, the Netherlands, France, and Belgium). While these EU countries increased their export to China, Hong Kong SAR, and other Asian economies (e.g., Malaysia and India), much of their remaining trade still took place within the EU. By contrast, for the USA and Japan, their top recipients included China, Hong Kong SAR, and their respective neighbors (i.e., Canada and Mexico for the US, and South Korea for Japan). Both of them also shipped a substantial amount of plastic waste to other Asian countries including Malaysia, Thailand, Vietnam, Indonesia, and the Philippines.

The 2019 trade network looks drastically different from previous years, as China’s ban on plastic waste imports indeed became a game changer. Although large flows into China vanished, the country remained visible on the map, as it still received imports from a fairly large number of trading partners (all in small amount though). Hong Kong SAR’s imports also declined significantly, and it reexported much of its imports to Southeast Asian countries (e.g., Thailand, Malaysia, Vietnam, Indonesia, and the Philippines). Malaysia and Thailand became the top two receivers, with major flows from the USA, Japan, Hong Kong SAR, the UK, and Germany. The imports by Turkey, India, Indonesia, and Vietnam also increased substantially. For Indonesia, one interesting finding was that the small island state the Marshall Islands became the country’s largest supplier. As for the global sources, the volume of trade decreased for almost all the trading territories. Except for Hong Kong SAR, all the top ten exporters were Annex VII members. For major EU countries, trading within the EU remained a very large part of their trade.

## 5. Discussion

Does global waste trade represent toxic colonialism? An impeccable search for new value? Or a plausible move toward sustainability? Unfortunately, there does not appear to be a simple “yes” or “no” answer to these questions. Indeed, with a large volume of plastic waste (especially low-value, hard-to-recycle plastics) flowing from developed to developing nations, the main challenge of international environmental injustice has not changed. Nevertheless, unlike before, when poor nations accepted hazardous waste from rich countries in exchange for much needed foreign currency, entrepreneurs in developing countries are now actively buying plastic waste (and many other types of waste) for recycling business to make profit. The economic logic behind dumping waste in lowest-wage countries, which used to be considered “impeccable” for high-income countries [71], also became increasingly acceptable and even attractive for receiving countries (at least for the recycling industry). In large part, the transboundary trade in plastic waste in recent decades has been a market-driven search for new value by parties in both sending and receiving countries.

Over time, however, the transboundary trade in plastic waste has exacerbated pollution and marine litter in some major receiving countries where solid waste mismanagement is a challenging issue. Facing mounting environmental challenges, many developing countries no longer see waste as a resource or revenue-generator. After China banned the imports of foreign waste, many developing counties have started to introduce restrictions or bans out of similar environmental concerns. Southeast Asian countries such as Malaysia, the Philippines, and Indonesia have already started sending waste shipments back where they came from. The PIC procedure in international agreements and recent amendments to the Basel Convention will empower them to reject illicit or unwanted inflows of plastic.

Given the changing circumstances, the global plastic waste trade will likely continue to decline. There are increasing calls from environmental organizations and the media for there to be a ban on the transboundary trade in plastic waste. If in place, a ban on the international plastic waste trade will help alleviate pollution and health concerns in some developing counties. It will also likely lead to an increase in the production of virgin plastics in developing countries, which will also cause much more used plastics to end up in landfills or the natural environment in developed countries.

In the long run, the CE needs to be considered as a solution for resource scarcity, waste management and pollution control, and sustaining economic growth. Recent discussions of the CE point to the 3R approach—reduce, reuse, and recycle—that needs to be adopted to tackle the plastic pollution crisis and achieve sustainable development. To way to address plastic pollution, first and foremost, is to limit the amount of plastic produced and reduce an individual’s or society’s use of plastic products. Policy makers at different levels should reckon with building impactful public policy that is conducive to conservation. An increasing number of countries, territories, and localities are taking actions to ban or tax the use of disposable or single-use plastic products, such as plastic bags, plastic straws, and non-recyclable single-use plastic containers. This should be adopted as a universal ban. There is also a need for policy interventions focusing on the production (manufacturing and packaging) processes that produce or use plastic materials. The OECD recommends that countries tax the use of virgin plastics and reform the support for the production and consumption of fossil fuels [72].

Recycling and reusing, facilitated by trade, are important in reducing waste, curbing the demand for virgin plastics, and making a more efficient use of resources. Countries and localities need to expand or upgrade their recycling capacities to better recover the value and reduce litter and pollution. While the insufficient sorting and collection of infrastructure is often cited as a contributor to plastic waste accumulation and pollution in developing regions, it is causing the low availability of sorted waste to reach recyclers in developed regions like the EU [73]. The trade in waste and the scrap for recovery, including the transboundary trade, is an integral part of the CE model and a part of the solution to achieve sustainability.

The future of the global plastic waste trade lies in a tighter regulation and a better enforcement, as well as a closer cooperation among countries. The Basel Convention needs to develop clear mechanisms for the enforcement of the PIC procedure. To effectively regulate the exports and imports of plastic waste and scraps, countries party to the Basel Convention need to transform the provisions of the Basel Convention into national law (countries like the UK, Norway, Singapore, etc., have started working on this). In 2021, the European Commission proposed new rules about the transport of waste, including plastic waste, to ensure that the EU does not export waste that is difficult or too expensive to recycle, tackling waste trafficking more effectively by ensuring that it only exports what is permitted if the recipient countries can guarantee that the waste will be processed responsibly [74]. Other plastic waste exporting countries should follow this lead. Exporting countries may also partner with potential importers to build waste and scrap processing facilities in designated areas in the receiving country. As Yang (2020) argues, a better alternative to the Basel Convention’s current trade minimization approach based on an arbitrary North–South dichotomy may be an approach that encourages capable countries to import waste and help build a processing capacity for intended waste importers [75].

## 6. Conclusions

This paper has examined the changing geography of the global trade in plastic waste. The research reveals that the transboundary trade in plastic waste has grown significantly and become increasingly complex over time. For over two decades, China was the single largest receiver until very recently. Hong Kong SAR was another extremely important player, playing a “middleman” role between mainland China and the rest of the world. China’s ban on plastic waste imports has led to a redrawing of the map of global trade, with Hong Kong SAR’s role and trade status being greatly influenced, and more trade flows being directed to countries in Southeast Asia, South Asia, and Turkey (a less wealthy Annex VII member). Developed nations including the USA, Japan, Germany, the Netherlands, France, the UK, Belgium, Italy, etc., have been the largest exporters of plastic waste. Among them, for European countries, intra-EU trade flows accounted for a large part of their total trade.

The findings of the paper debunk the North–South or core–periphery dichotomy that is embedded in the GEJ tradition (including the EUE theory), as well as in international environmental regulatory regimes such as the Basel Convention. The movement of plastic waste from Annex VII to non-Annex VII territories has been the largest flow, but trade flows of other directions (e.g., within the North or the South), which have often been overlooked in the EUE literature and other GEJ traditions, turned out to be significant. There is a small but growing flow of plastic waste from non-Annex VII to Annex VII territories. These findings call into question the grouping of countries into Annex VII and non-Annex VII signatories in the Basel Convention as well as the primary goal of the Ban Amendment to restrict the waste flow from the former to the latter.

The paper contributes to the discussions about value that are central in political economic approaches to global trade by demonstrating the relative, spatial, and dynamic nature of the concept. Value is a relative concept in the sense that waste means different things in different countries and carries different weights in their economies. Post-consumer-used plastics, especially when mixed with other waste, are largely considered to be valueless, recalcitrant waste in developed countries due to the high labor and environmental costs associated with their recovery and reuse. In some newly industrializing economies where there is a booming recycling industry and an ever-increasing need for resources, plastic waste and scraps becomes a profitable commodity and valuable feedstock. Related to the relativeness of value is the spatiality of the concept. The interest and ability to recover value from used things is geographically uneven. It is that spatiality, or geographically differentiated ability, that enables and drives the global plastic waste trade network to operate. Meanwhile, we must not forget about the temporal and dynamic nature of value. The value of waste and scraps changes over time. It could also change when the country’s domestic circumstances change.

## Figures and Tables

**Figure 3:**
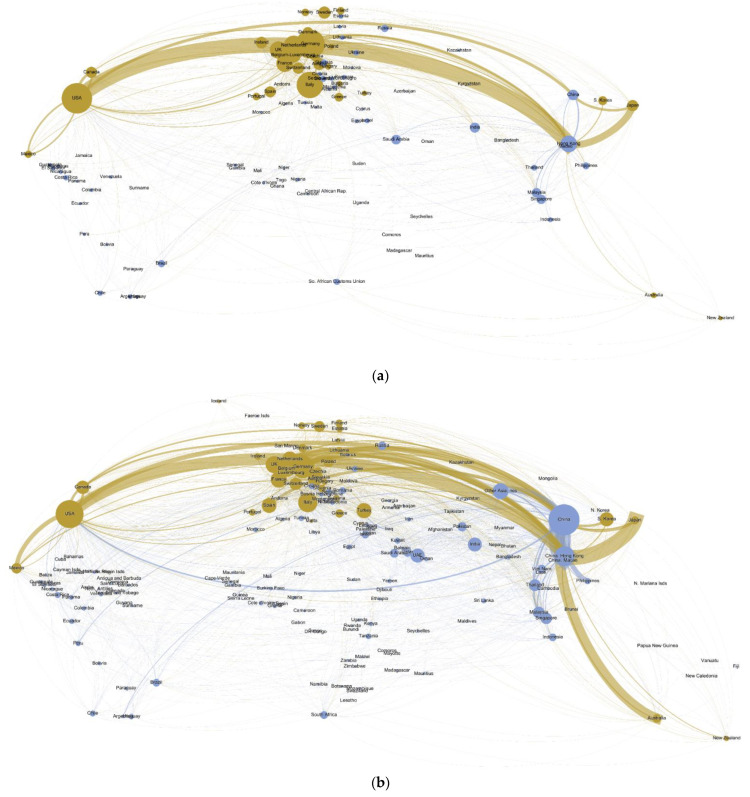

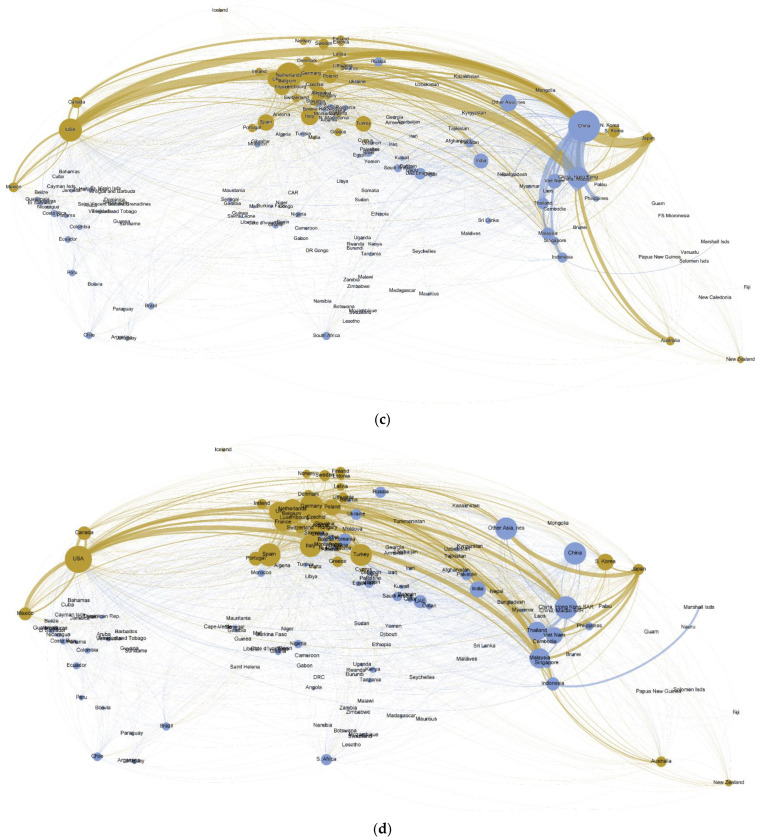
(**a**) Global trade network of plastic waste, 1996. (**b**) Global trade network of plastic waste, 2006. (**c**) Global trade network of plastic waste, 2016. (**d**) Global trade network of plastic waste, 2019.

## Data Availability

The data used in this paper mainly come from the UN Comtrade Database.
